# The Association among Literacy, Numeracy, HIV Knowledge and Health-Seeking Behavior: A Population-Based Survey of Women in Rural Mozambique

**DOI:** 10.1371/journal.pone.0039391

**Published:** 2012-06-22

**Authors:** Philip J. Ciampa, Lara M.E. Vaz, Meridith Blevins, Moshin Sidat, Russell L. Rothman, Sten H. Vermund, Alfredo E. Vergara

**Affiliations:** 1 Vanderbilt Institute for Global Health, Vanderbilt University School of Medicine, Nashville, Tennessee, United States of America; 2 Friends in Global Health, LLC, Maputo, Mozambique; 3 Department of Community Health, Universidade Eduardo Mondlane, Maputo, Mozambique; 4 Division of General Internal Medicine and Public Health, Vanderbilt University, Nashville, Tennessee, United States of America; Indiana University, United States of America

## Abstract

**Background:**

Limited literacy skills are common in the United States (US) and are related to lower HIV knowledge and worse health behaviors and outcomes. The extent of these associations is unknown in countries like Mozambique, where no rigorously validated literacy and numeracy measures exist.

**Methods:**

A validated measure of literacy and numeracy, the Wide Range Achievement Test, version 3 (WRAT-3) was translated into Portuguese, adapted for a Mozambican context, and administered to a cross-section of female heads-of-household during a provincially representative survey conducted from August 8 to September 25, 2010. Construct validity of each subscale was examined by testing associations with education, income, and possession of socioeconomic assets, stratified by Portuguese speaking ability. Multivariable regression models estimated the association among literacy/numeracy and HIV knowledge, self-reported HIV testing, and utilization of prenatal care.

**Results:**

Data from 3,557 women were analyzed; 1,110 (37.9%) reported speaking Portuguese. Respondents’ mean age was 31.2; 44.6% lacked formal education, and 34.3% reported no income. Illiteracy was common (50.4% of Portuguese speakers, 93.7% of non-Portuguese speakers) and the mean numeracy score (10.4) corresponded to US kindergarten-level skills. Literacy or numeracy was associated (p<0.01) with education, income, age, and other socioeconomic assets. Literacy and numeracy skills were associated with HIV knowledge in adjusted models, but not with HIV testing or receipt of clinic-based prenatal care.

**Conclusion:**

The adapted literacy and numeracy subscales are valid for use with rural Mozambican women. Limited literacy and numeracy skills were common and associated with lower HIV knowledge. Further study is needed to determine the extent to which addressing literacy/numeracy will lead to improved health outcomes.

## Introduction

In 2009 there were an estimated 22.5 million individuals living with HIV infection in sub-Saharan Africa and 1.8 million new infections. [1] Only 37% of those living in sub-Saharan Africa who were eligible for treatment were receiving antiretroviral therapy as of 2009, [1] and only 54% of pregnant women with HIV infection and 20% of their infants received drugs to prevent mother-to-child transmission. While these data indicate marked progress since 2000, there is considerable variation in coverage from country to country. [2] Factors that may explain these disparities have not been well-described, and developing a better understanding of these contributing factors may help to generate new strategies to improve HIV care across resource-limited settings. [3], [4]

Literacy skills include an individual’s ability to read, write and comprehend written language (print literacy), speak and understand spoken language (oral literacy) and understand and use numbers in daily life (quantitative literacy or numeracy). [5], [6] Limited literacy, defined as an ability to complete only basic, concrete, and everyday literacy-related tasks, is common in developed nations such as the United States (US), affecting over 100 million people. [7], [8] There is a growing body of evidence suggesting that literacy is an important independent factor to explain disparities in many health-related behaviors and outcomes. [9] Among individuals with HIV infection who live in the US, poor literacy skills have been associated with worse HIV knowledge, [10]–[13] lower adherence to antiretroviral treatment (ART), [14]–[15] and lower likelihood of achieving undetectable viral loads, [10]–[13] compared to those with adequate literacy, even after accounting for educational level. Individuals with limited literacy have more difficulty accessing and utilizing health care, communicating with providers, and performing self-care activities, potentially explaining the relationship between limited literacy and disparities in health knowledge, behaviors and outcomes. [16] The impact of limited literacy skills on health behaviors and outcomes may be amplified in settings where educational access and quality are variable and the prevalence of limited literacy is high, and may represent a novel target for interventions to improve care. However, to date these relationships have not been extensively studied in resource-limited settings, in part due to the lack of rigorously validated, standardized literacy measures. [17]–[21]

There are currently no direct measures of general literacy and numeracy that have been validated and published in the literature for use in non-English speaking populations in sub-Saharan Africa. We sought to adapt a measure of general literacy and numeracy, the Wide-Range Achievement Test, version 3 (WRAT-3) [22] for use in a Portuguese speaking country, and validate the adapted measure for use in Zambézia Province, Mozambique in a representative cross-section of female head-of-households who took part in the 2010 Ogumaniha* - Strengthening Communities through Integrated Programming (SCIP) Project baseline survey. [23] We examined the psychometric properties of these adapted measures with the hypothesis that higher literacy and numeracy would be associated with younger age, more education, income, and other markers of socioeconomic status. In addition, we examined the prevalence of low literacy in this population and the relationship between literacy/numeracy and HIV knowledge, HIV testing, and prenatal care utilization.

## Methods

### Ethics Statement

The study protocol was reviewed and approved by the National Committee of Bioethics for Health in Mozambique and the Institutional Review Board of Vanderbilt University. Written informed consent was obtained for all study participants.

### Study Population, Survey Design and Sampling Strategy

The *Ogumaniha-*SCIP Project baseline survey was designed to measure a range of socioeconomic and health issues pertaining to women, children and families living in Zambézia Province, Mozambique. A provincially representative sample of 264 enumeration areas was selected, with probability proportional to size according to the most recent census; this method intends for all households to have equal probability of selection. Using topographic and satellite maps, survey teams divided the enumeration areas into four quadrants. Starting in their assigned quadrant, an interviewer would systematically approach the first four households for interview. Interviewers conducted the survey with female head-of-households, defined as the principal wife of the immediate family of the household, from August 8 to September 25, 2010. The survey was translated into Portuguese and the five languages most commonly spoken in the province (Cisena, Elomwe, Echuabo, Cinyanja, and Emakhuwa); all language versions were piloted prior to the start of the survey. Except for the literacy measure, all survey questions were orally administered in the respondent’s language of choice; responses were entered using a cell phone application. The overall response rate was 99% of all households approached. Data from all respondents were eligible for analysis, however respondents with missing data for literacy or numeracy were excluded.

**Table 1:** Demographics characteristics of 3557 female heads-of-households surveyed in August-September, 2010 in Zambézia Province, Mozambique: unweighted sample size (N), weighted means or percentages.

Demographic Characteristic	All Women	Portuguese Speaking Status		
	N = 3557	Yes	No	P-value
		N = 1110 (37.9%)	N = 2447 (62.1%)	
**Mean age**	31.2	28.5	33.0	<0.001
**Mean persons in household**	4.8	4.9	4.8	0.8
**Education, %**				
<1 year	44.6	15.4	62.4	<0.001
1–5 years	43.6	57.1	35.3	
>5 years	11.8	27.5	2.2	
**Monthly income, %**				
No income	34.3	24.2	40.4	<0.001
≤1,000 Meticais*	51.5	53.2	50.5	
>1000 Meticais*	14.2	22.6	9.2	
**Language used at home, %**				
Cisena	13.4	11.8	14.4	<0.001
Elomwe	41.0	42.7	40.0	
Echuabo	23.0	23.1	22.9	
Portuguese	7.2	18.0	0.5	
Other†	15.5	4.6	22.1	
**Electricity in home, %**	8.8	16.5	4.2	0.002
**Mean kilometers to health center**	7.7	6.6	8.4	0.01
**Mean HIV knowledge score** ‡	3.3	3.6	3.1	0.1

* 1,000 Meticais = approximately 34 dollars at the time of study (Aug-Sept 2010).

† Other includes: *Cinyanja, Emakhuwa, Xironga, Xitswa.*

‡ Scores range from 0–9.

Missing data: age (521/3557), income (277/3557), HIV knowledge (365/3557).

### Study Measures

The WRAT-3 is a validated, English language measure of general literacy and numeracy; [22] we adapted the word reading and arithmetic subscales. The word reading subscale consists of two lists of 15 letters and 42 words of escalating contextual and phonetic complexity. Participants attempt to read each item on the list and are scored for proper pronunciation. Scores range from 0–57; scores <16 indicate the ability to read individual letters only. Respondents where characterized as having low literacy if they had scores 0–15 (US grade level: kindergarten or less), intermediate literacy if their scores were 16–56, and high literacy if they had scores of 57, corresponding to the demonstrated ability to read the entire word list properly (57 is equivalent to post-high school US grade level). One word reading list was translated into Portuguese by a native speaker of Portuguese, modified to fit the vocabulary of Mozambique, and back-translated into English to ensure content fidelity. In one instance there was a discrepancy in the complexity of a word in Portuguese versus in English; a word of the equivalent level of complexity was substituted from the second word reading list. The oral portion of the WRAT arithmetic subscale was translated into Portuguese, adapted for cultural context, and back translated into English. It consists of 15 orally administered items augmented by a visual aid card; scores range from 0–15. A perfect score of 15 is the US equivalent of 1^st^ grade math skills. Both items were field tested prior to the start of data collection. The numeracy scale was administered in the respondent’s language of choice, and the literacy scale was administered in Portuguese only.

**Table 2:** General literacy and numeracy scores for 3557 female heads-of-households surveyed in Zambézia Province, Mozambique, during August-September, 2010: unweighted sample size (N), weighted means or proportions.

Literacy and Numeracy Skill	Total	Portuguese Speaking Status‡	
	N = 3557	Yes	No
		N = 1110	N = 2447
**Literacy score,** * **%**			
0–15	77.3	50.4	93.7
16–56	11.1	23.2	3.7
57	11.6	26.4	2.6
**Mean Numeracy Score** †	10.4	12.6	9.1
**Numeracy Item, % correct**			
1. Count 3 crocodiles	87.4	94.4	83.1
2. Count 5 boxes	85.5	94.7	79.9
3. Count 15 circles	78.0	87.5	72.2
4. Read 5 numbers	40.6	64.9	25.8
5. Hold up 3 fingers	89.2	94.6	85.9
6. Hold up 8 fingers	81.5	90.8	75.9
7. Which number is larger, 9 or 6?	72.7	87.8	63.5
8. Which number is larger 42 or 28?	60.7	80.1	48.8
9. If you have 3 Meticais and spend 1, how many do you have left?	79.5	91.0	72.5
10. How many are 3 mangos plus 4 mangos?	69.3	78.4	63.7
11. João had 9 pencils. He lost 3 of them. How many does he have left?	62.2	74.4	54.8

* Literacy scores ranged from 0 to 57, with lower scores corresponding to lower literacy.

† Numeracy scores ranged from 0–15, with lower scores indicating lower numeracy. Respondents were given 1 point per correct answer, except for item 4, where 1 point was given for each number read correctly.

‡ p<0.001 for all pair-wise comparisons between Portuguese speakers and non-Portuguese speakers.

Demographic data and Portuguese speaking ability were collected by participant self-report. Respondents who reported Portuguese as their native language or who responded “yes” to an item asking if they “spoke Portuguese well” were classified as Portuguese speakers. Distance from the center of the enumeration area to the nearest health center was calculated in kilometers using GPS coordinates. HIV knowledge was measured with five items adapted from Demographic Health Survey 5 that assess knowledge of HIV transmission, transmission prevention, and HIV treatment; [24] scores ranged from 0–9. Receipt of HIV testing and prenatal care were assessed by self-report.

**Table 3:** Relationship between literacy and selected sociodemographic data for 3557 Portuguese speaking and non-Portuguese speaking women in Zambézia Province, Mozambique during August-September, 2010.

Characteristic	Portuguese Speakers			Non-Portuguese Speakers			
	*N*	*Literacy Score (ρ)*	*P-value*	*N*		*Literacy Score (ρ)*	*P-value*
**Age**	1013	−0.14	<0.001		2023	−0.20	<0.001
**Years of education**	1110	0.70	<0.001		2447	0.50	<0.001
**Reading materials, #**	1108	0.21	<0.001		2446	0.13	<0.001
**HIV knowledge**	1109	0.27	<0.001		2183	0.14	<0.001
**Characteristic**	***N***	***Median Literacy Score (IQR)***	***P-value***		***N***	***Median Literacy Score (IQR)***	***P-value***
**Mobile phone in home**							
Yes	167	41 (2–57)	<0.001		111	0 (0–0)	0.4
No	595	10 (0–57)			1673	0 (0–0)	
**Electricity in home**							
Yes	112	57 (22–57)	<0.001		36	0 (0–5.5)	<0.001
No	997	8 (0–57)			2406	0 (0–0)	
**Bank account**							
Yes	86	57 (35–57)	<0.001		30	0 (0–2)	0.2
No	1019	10 (0–57)			2411	0 (0–0)	
**Roofing material**							
Straw/Cane/Grass	914	9 (0–57)	<0.001		2248	0 (0–0)	0.5
Metal/Cement	174	48.5 (2–57)			169	0 (0–0)	
**Monthly income**							
No income	329	7 (0–57)	<0.001		1196	0 (0–0)	<0.001
≤1000 Meticais	535	15 (0–57)			881	0 (0–0)	
>1000 Meticais	164	45 (1.5–57)			175	0 (0–4)	
**Transportation to town**							
Foot	261	10 (0–57)	0.001		724	0 (0–0)	0.001
Bike	279	5 (0–46)			710	0 (0–0)	
Car/motorcycle/bus	510	36.5 (0–57)			730	0 (0–2)	
Other	48	0 (0–12.5)			254	0 (0–0)	
**Language used at home**							
Cisena	222	0 (0–7)	<0.001		1009	0 (0–0)	<0.001
Echuabo	329	2 (0–38)			613	0 (0–0)	
Elomwe	452	50 (5–57)			659	0 (0–10)	
Portuguese	91	57 (16–57)			9	48.5 (0–57)	
Other	16	31.5 (0–53)			152	0 (0–0)	

### Data Analysis

Summary statistics are reported for demographics, literacy score category, and numeracy as unweighted sample size, and weighted means or proportions. The weighted proportions of correct responses for each numeracy item are also reported. Estimates of descriptive statistics were weighted by the inverse of the household sampling probability. All data analyses were stratified by self-reported Portuguese speaking status; weighted summary data were compared for Portuguese speakers and non-Portuguese speakers by adjusted Wald test for mean values and Pearson chi-squared test for proportions.

To evaluate the construct validity of the adapted literacy and numeracy measures, we examined the association of literacy and numeracy scores with selected characteristics. We hypothesized *a priori* that lower literacy or numeracy would be associated with higher age, less education and income, fewer reading materials in the home and household assets, non-motorized transportation, languages other than Portuguese spoken at home; these associations were tested by Spearman rank correlation, Wilcoxon rank sum and Kruskal-Wallis tests, as statistically appropriate. Effects of clustering were not considered in construct validation analyses. Internal consistency reliability was tested by Chronbach’s alpha for the HIV knowledge items and Kuder-Richardson 20 for numeracy items.

**Table 4:** The association among literacy or numeracy, HIV knowledge, and sociodemographic characteristics for 3557 women in Zambézia Province, Mozambique during August-September, 2010.

HIV Knowledge	Portuguese Speakers						Non-Portuguese Speakers			
	N		β	95% CI	P-value	N		β	95% CI	P-value
***Literacy Score***										
** 0** *–* **15**		**417**	**–**			**1534**		**–**		
** 16** *–* **56**		**146**	**1.04**	**0.31, 1.76**	**0.005**	**71**		**0.31**	**−1.26, 1.87**	**0.7**
** 57**		**279**	**1.10**	**0.34, 1.86**	**0.005**	**76**		**1.35**	**0.48, 2.22**	**0.003**
* Age (per year)*		842	0.0	−0.02, 0.02	1.0	1681		−0.01	−0.03, 0.01	0.4
* Distance to health facility (per km)*		842	−0.10	−0.13, −0.06	<0.001	1681		0.0	−0.05, 0.05	0.9
* Income*										
* No income*		257	**–**			878		**–**		
≤1000 meticais		445	0.25	−0.43, 0.93	0.5	650		1.78	1.26, 2.29	<0.001
>1000 meticais		140	1.16	0.49, 1.83	0.001	153		1.38	0.54, 2.21	0.001
* Survey Language*										
* Portuguese*		110	**–**			12		**–**		
* Cisena*		141	−0.27	−1.08, 0.54	0.5	681		−0.24	−0.85, 0.37	0.4
* Echuabo*		247	−0.20	−0.76, 0.36	0.5	410		−0.26	−1.17, 0.65	0.6
* Elomwe*		336	0.09	−0.48, 0.67	0.8	454		0.27	−0.58, 1.12	0.5
* Other*		8	−1.41	−2.18, −0.64	<0.001	124		0.63	−0.1, 1.36	0.09
* Education*										
* <1 year*		111	**–**			1000		**–**		
* 1–5 years*		506	−0.17	−0.71, 0.36	0.5	638		−0.36	−0.93, 0.21	0.2
* >5 years*		225	0.02	−0.69, 0.73	1.0	43		1.42	−0.22, 3.06	0.09
***Numeracy Score (per 1 point increase)***		**842**	**0.14**	**0.03, 0.24**	**0.01**	**1681**		**0.18**	**0.09, 0.26**	**<0.001**
* Age (per year)*		842	0.01	−0.02, 0.03	0.6	1681		0.0	−0.03, 0.2	0.7
* Distance to health facility (per km)*		842	−0.09	−0.12, −0.05	<0.001	1681		0.0	−0.05, 0.05	0.9
* Income*										
* No income*		257	**–**			878		**–**		
≤1000 meticais		445	0.26	−0.35, 0.87	0.4	650		1.55	0.97, 2.13	<0.001
>1000 meticais		140	1.33	0.71, 1.96	<0.001	153		1.11	0.33, 1.9	0.01
* Survey Language*										
* Portuguese*		110	**–**			12		**–**		
* Cisena*		141	−0.52	−1.21, 0.17	0.1	681		0.0	−0.82, 0.83	1.0
* Echuabo*		247	−0.18	−0.73, 0.38	0.5	410		0.18	−1.02, 1.37	0.8
* Elomwe*		336	0.09	−0.54, 0.72	0.8	454		0.72	−0.33, 1.77	0.2
* Other*		8	−1.75	−2.64, −0.86	<0.001	124		0.57	−0.50, 1.64	0.3
* Education*										
* <1 year*		111	**–**			1000		**–**		
* 1–5 years*		506	−0.28	−1.02, 0.47	0.5	638		−0.73	−1.42, −0.05	0.04
* >5 years*		225	0.21	−0.70, 1.12	0.7	43		1.19	−0.32, 2.7	0.1

Multivariable linear regression models accounting for the effects of clustering by enumeration area were used to examine the relationship of literacy or numeracy and HIV knowledge; logistic regression was used to examine the relationship between literacy or numeracy and receipt of antenatal care and HIV testing. A second set of multivariable regression models adjusted for important covariates identified *a priori*, including: respondent age, reported income, language of survey administration, and distance from health center (km). A third set added education into the adjusted model. All analyses were conducted using STATA™ statistical software package (STATAcorp, Release 11, College Station, TX).

**Table 5:** Relationship between numeracy and selected sociodemographic data for 3557 Portuguese speaking and non-Portuguese speaking women in Zambézia Province, Mozambique during August-September, 2010.

Characteristic		Portuguese Speakers				Non-Portuguese Speakers		
	*N*		*Numeracy Score (ρ)*		*P-value*	*N*	*Numeracy Score (ρ)*	*P-value*
**Age**	1013		−0.13		<0.001	2023	−0.22	<0.001
**Years of education**	1110		0.58		<0.001	2447	0.41	<0.001
**Reading materials, #**	1108		0.21		<0.001	2446	0.25	<0.001
**Literacy score**	1110		0.66		<0.001	2447	0.40	<0.001
**HIV knowledge**	1109		0.29		<0.001	2183	0.28	<0.001
**Characteristic**	***N***		***Median Numeracy Score (IQR)***		***P-value***	***N***	***Median Numeracy Score (IQR)***	***P-value***
**Mobile phone in home**								
Yes	167		15 (12–15)	<0.001		111	10 (8–13)	0.02
No	595		14 (10–15)			1673	9 (6–12)	
**Electricity in home**								
Yes	112		15 (14–15)	<0.001		36	10 (7.5–14)	0.1
No	997		14 (11–15)			2406	9 (6–13)	
**Bank account**								
Yes	86		15 (15–15)	<0.001		30	10 (7–11)	0.8
No	1019		14 (11–15)			2411	9 (6–13)	
**Roofing material**								
Straw/Cane/Grass	914		14 (11–15)	<0.001		2248	9 (6–12)	0.01
Metal/Cement	174		15 (13–15)			169	10 (7–13)	
**Monthly income**								
No income	329		13 (10–15)	<0.001		1196	9 (5–11)	<0.001
≤1000 Meticais	535		15 (12–15)			881	10 (7–14)	
>1000 Meticais	164		15 (12–15)			175	11 (8–14)	
**Transportation to town**								
Foot	261		14(10–15)	<0.001		724	8 (5–11)	<0.001
Bike	279		13(10–15)			710	10(7–12)	
Car/motorcycle/bus	510		15(12–15)			730	10(6–15)	
Other	48		12.5(10–15)			254	8(3–12)	
**Language used at home**								
Cisena	222		11 (9–15)	<0.001		1009	9 (6–11)	<0.001
Echuabo	329		13 (10–15)			613	7 (4–10)	
Elomwe	452		15 (13–15)			659	12 (7–15)	
Portuguese	91		15 (14–15)			9	15 (11–15)	
Other	16		13.5 (11–15)			152	12 (10–15)	

## Results

### Sociodemographic Characteristics

We interviewed 3,636 female heads-of-households. Case-wise deletion of participants with missing responses excluded 33 missing literacy and 46 missing numeracy such that 3,557 respondents (98%) were included in analyses. Women had a mean age of 31.2 years; 37.9% reported that they spoke Portuguese well and were classified as Portuguese-speakers (**Table 1**). Even for women who reported Portuguese speaking ability, most spoke a language other than Portuguese at home (82.0%). Among all women, 44.6% had no formal education and only 11.8% had >5 years of education. No income was reported for 34.3% of women, and only 14.2% reported a monthly income of >1,000 *Meticais* ($34 USD). The mean distance to the nearest health facility was 7.7 km (range: 0.2–37.3 km). The mean HIV knowledge score was 3.3 (range: 0–9); alpha was 0.75 for these items. Respondents who reported the ability to speak Portuguese were younger, had more education and higher income, were more likely to have electricity in their home and lived closer to a health center than those who did not speak Portuguese.

### General Literacy Findings

Over half (50.4%) of Portuguese speakers and nearly all (93.7%) non-Portuguese speakers scored in the lowest category of literacy, corresponding to either no reading ability or the ability to read individual letters only. A perfect score of 57 was demonstrated by 26.4% of Portuguese speakers and 2.6% of non-Portuguese speakers (**Table 2**). For both Portuguese and non-Portuguese speaking respondents, higher literacy scores were correlated with younger age (Portuguese speakers [PS]: ρ = −0.14, p<0001; non-Portuguese speakers [NPS]: ρ = −0.20, p<0.001), more education (PS: ρ = 0.70, p<0001; NPS: ρ = 0.50, p<0.001), more reading materials in the home (PS: ρ = 0.21, p<0001; NPS: ρ = 0.13, p<0.001) and higher HIV knowledge (PS: ρ = 0.27, p<0001; NPS: ρ = 0.14, p<0.001). Among the Portuguese speaking respondents, those who reported more income, used motorized transportation, owned a house with a metal or cement roof, owned a cell phone, had bank accounts or electricity, and spoke Portuguese at home had higher median literacy scores than those with fewer assets or who spoke another language primarily at home. For non-Portuguese speakers, there was a statistically significant association between higher median literacy and having a home with electricity, having higher income, and using motorized transportation; however literacy scores for non-Portuguese speakers as a whole were low for the non-Portuguese speaking group (**Table 3**).

For both Portuguese and non-Portuguese speakers, literacy was independently associated with HIV knowledge after adjusting for respondent age, stated income, distance to the nearest health center, and the language of the survey in models with and without an adjustment for education; for non-Portuguese speakers this association was observed only for those with the highest literacy scores (**Table 4**). We did not detect significant associations between literacy and self-reported HIV testing or prenatal care attendance.

### General Numeracy Findings

The Kuder-Richardson coefficient for numeracy items was 0.84 for Portuguese speakers and 0.87 for non-Portuguese speakers. Mean numeracy scores were higher for Portuguese speakers (12.6) than for non-Portuguese speakers (9.1); performance for each item of the numeracy scale is summarized in **Table 2**. Fewer respondents correctly answered items of greater vs. lesser complexity for a given task; for instance, while 91.0% of Portuguese speaking respondents and 72.5% of non-Portuguese speakers correctly answered an item that tests subtraction of three minus one, only 74.4% of Portuguese speakers and 54.8% of non-Portuguese speakers correctly answered an item that tested subtraction of nine minus three.

For both Portuguese and non-Portuguese speaking respondents, higher numeracy scores were correlated with younger age (PS: ρ = −0.13, p<0001; NPS: ρ = −0.22, p<0.001), more education (PS: ρ = 0.58, p<0001; NPS: ρ = 0.41, p<0.001), more reading materials in the home (PS: ρ = 0.21, p<0001; NPS: ρ = 0.25, p<0.001) more HIV knowledge (PS: ρ = 0.29, p<0001; NPS: ρ = 0.28, p<0.001) and with higher literacy scores (PS: ρ = 0.66, p<0001; NPS: ρ = 0.40, p<0.001). Portuguese and non-Portuguese speaking respondents who reported more income, used motorized transportation, owned a house with a metal or cement roof, owned a cell phone and spoke Portuguese at home had higher median numeracy scores compared to their counterparts. For Portuguese speaking respondents, individuals who reported having bank accounts or electricity in their home had higher median numeracy scores than those who lacked them; there was no such association among non-Portuguese speaking respondents (**Table 5**).

In multivariable linear regression, numeracy was significantly associated with HIV knowledge independent of respondent age, income, distance from the hospital and the language of survey administration; this association remained significant with education included in the model (**Table 4**). We did not detect significant associations between numeracy and self-reported HIV testing or prenatal care attendance.

## Discussion

In this provincially representative sample of 3,557 women, the adapted WRAT-3 was reliable and valid for measuring general literacy and numeracy. Poor literacy and numeracy were common for women living in Zambézia Province, and similarly many women had difficulty with items that assessed basic numerical concepts and computational skills. For both Portuguese speakers and non-speakers, poorer literacy and numeracy skills were associated with less HIV knowledge but not lower self-reported utilization of HIV testing or prenatal care. To our knowledge, this is the first study in the literature that reports directly measured literacy and numeracy skill in a representative population in rural sub-Saharan Africa. Our use of representative data to estimate the literacy and numeracy skills for all female heads-of-household in the province is a major strength of this study.

The prevalence of illiteracy in our study is substantial, and higher than the recent estimate of the Mozambican national literacy rate of 54%. [25] We assessed literacy in a group of women living in predominantly rural settings who had a low level of formal education, likely explaining these differences. This study builds on other work done in sub-Saharan Africa to estimate literacy by utilizing a direct test of individual literacy and numeracy skill, rather than estimating literacy indirectly by self-report, [17] national census data, [18] or directly by use of non-standardized measures of reading comprehension. [19], [21] One study adapted a validated English language test of health literacy, the Rapid Estimation of Adult Literacy in Medicine (REALM) to measure health literacy in 125 bilingual South Africans, but concluded that its use was unsuitable because poor comprehension of many of the included health terms. [20] Based on our data from Zambézia, the development of language and context appropriate health literacy or functional literacy measurement tools should be a topic for further work in low and middle income settings.

The deficits in numeracy for many women in this study are stark, and may have serious implications for behaviors necessary for utilization of health care for themselves and their children. To our knowledge, previous to this study there was no measure published in the literature estimating numeracy by direct measurement in persons living in sub-Saharan Africa. Work in the US looking at literacy and medication administration have shown that individuals with low literacy have difficulty appropriately dosing liquid medications for their children [26]–[28] and with managing their own medications. [10], [29] These challenges, if generalizable to HIV-affected women in Zambézia for example, may be even more important given the potential harm from too much or too little antiretroviral medication given during prevention of mother-to-child transmission care, self-care, and assistance to family members, notably treatment to children.

The positive associations of literacy and numeracy with HIV knowledge are similar to evidence from US studies among individuals living with HIV infection. [10]–[13] While the literacy skills measured in this study may or may not be directly required for interaction with the health system, there is evidence that individuals with low literacy have difficulty communicating with health-care providers. [30] This may be a particular problem in health care settings such as those in Zambézia, where often the language of the provider (often Portuguese) differs from the local languages in which patients have fluency and use as their primary language at home. [3], [4], [31] The lack of association between literacy and health seeking behavior may reflect that among the multitude of barriers facing women in accessing care in rural Zambézia, literacy skills are less important to access care *per se*. The use of self-reported outcomes may also limit the ability to examine these associations, as individuals with poor literacy may be more likely to be unaware of past HIV testing.

In addition to the limitation of our use of a general literacy scale in this study (vs. a functional scale), there are potential methodological limitations with using a word list for measuring literacy in the Portuguese language. The WRAT-3 reading list uses pronunciation of words as a proxy measurement for reading comprehension; while rapid to administer and valid, this approach may overestimate literacy. [32] This effect has been shown to be more pronounced when using these types of literacy measures in languages such as Spanish, where there is a direct relationship between the appearance and pronunciation of a given word. [32]–[34] One study that attempted to adapt a word-reading literacy test into Spanish was successful only in generating dichotomous groups of high or low literacy. [34] Other limitations to this study include the cross-sectional design of the study and the use of self-reported data, making some of our findings potentially subject to confounding and recall bias. The use of a non-validated HIV knowledge scale and the fact that ten-percent of participants did not respond to the HIV-knowledge items introduces the possibility for misclassification bias. The study included data from women living in one province in rural Mozambique only, and so results may not be applicable to the male population, persons living in other Provinces, or to individuals living in more urban settings.

Our study introduces valid instruments to measure general numeracy and literacy that could be adapted for other settings and languages, and also demonstrates clearly some of the skill disparities facing women within one rural province in Mozambique. These data suggest that literacy and numeracy may be important independent factors in the acquisition of HIV knowledge, and have implications for public health efforts in rural settings such as Zambézia where literacy and numeracy skills are limited. The level of literacy and numeracy skill should be taken into consideration when developing HIV educational messages in resource-limited settings such as Zambézia. While efforts to improve primary school education access and quality for women is clearly a high national priority, interventions designed to improve the quality of provider health communication or improve the applied literacy and numeracy skills of patients during HIV care and treatment programs may be simple, inexpensive and have a more immediate impact. [35], [36] A better understanding of how literacy and numeracy skills are related to health behaviors, skills and knowledge would inform efforts to overcome health disparities and may generate new strategies to improve health care quality in settings where low literacy is common.

## References

[1] UNAIDS (2010). Global Report: UNAIDS report on the global AIDS epidemic 2010.. Geneva, Switzerland: Joint United Nations Programme on HIV/AIDS (UNAIDS).

[2] World Health Organization (2011). HIV in the WHO African region: Progress towards achieving universal access to priority health sector interventions 2011 update.. http://www.afro.who.int/en/clusters-a-programmes/dpc/acquired-immune-deficiency-syndrome/features/3015-hiv-in-the-who-african-region-progress-towards-achieving-universal-access-to-priority-health-sector-interventions-2011-update.html.

[3] Audet CM, Burlison J, Moon TD, Sidat M, Vergara AE (2010). Sociocultural and epidemiological aspects of HIV/AIDS in Mozambique.. BMC Int Health Hum Rights.

[4] Groh K, Audet CM, Baptista A, Sidat M, Vergara A (2011). Barriers to antiretroviral therapy adherence in rural Mozambique.. BMC Public Health.

[5] Institute of Medicine (2004). Health literacy: a prescription to end confusion.. Washington DC: National Academies Press.

[6] Rothman RL, Montori VM, Cherrington A, Pignone MP (2008). Perspective: the role of numeracy in health care.. J Health Commun.

[7] Kutner M, Greenberg E, Jin Y, Paulsen C (2006). The Health Literacy of America’s Adults: Results From the 2003 National Assessment of Adult Literacy.. Washington, DC: U.S. Department of Education, National Center for Education Statistics.

[8] Kutner M, Greenberg E, Baer J (2005). A First Look at the Literacy of America’s Adults in the 21st Century.. Washington, DC: National Center for Education Statistics.

[9] Berkman ND, Sheridan SL, Donahue KE, Halpern DJ, Crotty K (2011). Low health literacy and health outcomes: an updated systematic review.. Ann Intern Med.

[10] Kalichman SC, Benotsch E, Suarez T, Catz S, Miller J (2000). Health literacy and health-related knowledge among persons living with HIV/AIDS.. Am J Prev Med.

[11] Kalichman SC, Rompa D (2000). Functional health literacy is associated with health status and health-related knowledge in people living with HIV-AIDS.. J Acquir Immune Defic Syndr.

[12] Wolf MS, Davis TC, Cross JT, Marin E, Green K (2004). Health literacy and patient knowledge in a Southern US HIV clinic.. Int J STD AIDS.

[13] Wolf MS, Davis TC, Arozullah A, Penn R, Arnold C (2005). Relation between literacy and HIV treatment knowledge among patients on HAART regimens.. AIDS Care.

[14] Kalichman SC, Ramachandran B, Catz S (1999). Adherence to combination antiretroviral therapies in HIV patients of low health literacy.. J Gen Intern Med.

[15] Miller LG, Liu H, Hays RD, Golin CE, Ye Z (2003). Knowledge of antiretroviral regimen dosing and adherence: a longitudinal study.. Clin Infect Dis.

[16] Osborn CY, Paasche-Orlow MK, Bailey SC, Wolf MS (2011). The mechanisms linking health literacy to behavior and health status.. Am J Health Behav.

[17] Hegazi A, Bailey RL, Ahadzie B, Alabi A, Peterson K (2010). Literacy, education and adherence to antiretroviral therapy in The Gambia.. AIDS Care.

[18] McTavish S, Moore S, Harper S, Lynch J (2010). National female literacy, individual socio-economic status, and maternal health care use in sub-Saharan Africa.. Soc Sci Med.

[19] Underwood C, Serlemitsos E, Macwangi M (2007). Health communication in multilingual contexts: a study of reading preferences, practices, and proficiencies among literate adults in Zambia.. J Health Commun.

[20] Dowse R, Lecoko L, Ehlers MS (2010). Applicability of the REALM health literacy test to an English second-language South African population.. Pharm World Sci.

[21] Stuebing KW (1997). Maternal schooling and comprehension of child health information in urban Zambia: is literacy a missing link in the maternal schooling-child health relationship?. Health Transit Rev.

[22] Wilkinson GS (1993). Wide range achievement test: Administration manual.. Willmington, Del: Wide Range, Inc.

[23] Vergara A, Blevins M, Vaz L, Manders E, Olupona O (2011). Baseline survey report for SCIP – Ogumaniha: Improving health and livelihoods of children, women and families in the Province of Zambézia, Republic of Mozambique.. http://www.globalhealth.vanderbilt.edu/community-andservice/SCIP/scip_docs/report_20110531.pdf/view.

[24] ORCMacro (2006). Measure DHS: Model Questionnaire with Commentary.. Calverton, MD: DHS.

[25] United Nations Development Programme (2010). Human Development Report 2010, 20th Anniversary Edition: The Real Wealth of Nations: Pathways to Human Development.. New York, NY: UNDP.

[26] Yin HS, Mendelsohn AL, Wolf MS, Parker RM, Fierman A (2010). Parents’ medication administration errors: role of dosing instruments and health literacy.. Arch Pediatr Adolesc Med.

[27] Yin HS, Mendelsohn AL, Fierman A, van Schaick L, Bazan IS (2011). Use of a pictographic diagram to decrease parent dosing errors with infant acetaminophen: a health literacy perspective.. Acad Pediatr.

[28] Yin HS, Dreyer BP, Foltin G, van Schaick L, Mendelsohn AL (2007). Association of low caregiver health literacy with reported use of nonstandardized dosing instruments and lack of knowledge of weight-based dosing.. Ambul Pediatr.

[29] Kripalani S, Henderson LE, Chiu EY, Robertson R, Kolm P (2006). Predictors of medication self-management skill in a low-literacy population.. J Gen Intern Med.

[30] Doak C, Doak L, Root J (1996). Teaching Patients wiht Low-Literacy Skills (2d ed.).. Philadelphia, PA: JB Lippincott.

[31] Moon TD, Burlison JR, Sidat M, Pires P, Silva W (2010). Lessons Learned while Implementing an HIV/AIDs Care and Treatment Program in Rural Mozambique.. Retrovirol: Res Treatment.

[32] Davis TC, Michielutte R, Askov EN, Williams MV, Weiss BD (1998). Practical assessment of adult literacy in health care.. Health Educ Behav.

[33] Lee SY, Bender DE, Ruiz RE, Cho YI (2006). Development of an easy-to-use Spanish Health Literacy test.. Health Serv Res.

[34] Nurss J, Barker D, Davis T, Parker R, Williams M (1995). Difficulties in functional health literacy screening in Spanish-speaking adults.. J Reading.

[35] Ciampa PJ, Burlison JR, Blevins M, Sidat M, Moon TD (2011). Improving retention in the early infant diagnosis of HIV program in rural Mozambique by better service integration.. J Acquir Immune Defic Syndr.

[36] Cook RE, Ciampa PJ, Sidat M, Blevins M, Burlison J (2011). Predictors of successful early infant diagnosis of HIV in a rural district hospital in Zambezia, Mozambique.. J Acquir Immune Defic Syndr.

